# Population-based serology reveals risk factors for RSV infection in children younger than 5 years

**DOI:** 10.1038/s41598-021-88524-w

**Published:** 2021-04-26

**Authors:** Stijn P. Andeweg, Rutger M. Schepp, Jan van de Kassteele, Liesbeth Mollema, Guy A. M. Berbers, Michiel van Boven

**Affiliations:** Centre for Infectious Disease Control, National Institute for Public Health and the Environment (RIVM), Antonie van Leeuwenhoeklaan 9, 3720 BA Bilthoven, The Netherlands

**Keywords:** Microarrays, Risk factors, Viral infection, Paediatric research, Health policy

## Abstract

Respiratory syncytial virus (RSV) infection is a leading cause of hospitalization in infants. Underlying risk factors for RSV infection in the general population are not well understood, as previous work has focused on severe outcomes of infection in a clinical setting. Here we use RSV-specific IgG and IgA antibody measurements from two population-based cross-sectional serosurveys carried out in the Netherlands (n = 682) to classify children up to 5 years as seronegative or seropositive. We employ a generalized additive model to estimate the probability of prior RSV infection as function of age, date of birth within the year, and other risk factors. The analyses show that the majority of children have experienced a RSV infection before the age of 2 years. Age and birthdate are strong predictors of RSV infection in the first years of life, and children born in summer have higher estimated probability of infection than those born in winter [e.g., 0.56 (95% CI 0.45–0.66) vs. 0.32 (0.21–0.45) at age 1 year]. Our analyses reveal that the mean age at infection depends on date of birth, which has implications for the design of vaccination programmes and prioritisation schemes for the prophylactic use of monoclonal antibodies.

## Introduction

Respiratory syncytial virus (RSV) is a main cause of acute respiratory infection (ARI) in infants and young children^1^, leading to an estimated 60,000 in-hospital deaths and 3.2 million hospital admissions per year in children younger than 5 years^2^. In addition, RSV is also increasingly recognized as a main cause of the burden of respiratory disease in older adults^3^. In young children the overall disease burden depends critically on the age of primary infection, where prematurity and young age (< 6 months) are associated with severe disease^4^.

Despite the progress being made in vaccine and monoclonal antibody development^5^, there still is an incomplete quantitative understanding of the RSV infection dynamics across all age groups. Most research on RSV infections is done with a focus on severe outcome of infection in a clinical setting^6^. Building on such information, attempts have been undertaken to gauge the incidence of infection^4,7^ and infection attack rates^8^, showing that these are highest in young children^9^. Nevertheless, the majority of infections lead to relatively mild illness for which no medical care is sought. For a proper understanding of the transmission dynamics of RSV and optimal planning of preventative measures, direct information is needed on the incidence of infection in different age groups, irrespective of clinical signs. Such information on the cumulative incidence of infection can be obtained from serological surveys.

Here we study the infection dynamics of RSV in children under 5 years in the Netherlands by fitting a generalized additive model (GAM) to serological antibody data from two large cross-sectional population based studies^10,11^. Our analyses uncover risk factors for infection with RSV in the first years of life, providing quantitative estimates of the infection probabilities as function of relevant covariates. Hence, our analyses complement and extend on earlier studies that focussed on risk factors for severe outcome and mortality^12^.

## Methods

### Data

We use serological data from two large Dutch population-based cross-sectional seroprevalence studies carried out in 2006/2007 and 2016/2017^10,11^. We focus on infants and young children up to 60 months of age. The youngest participant in our study is 1 month old (36 days). In total, 450 individuals originated from the 2006/2007 cohort and 741 from the 2016/2017 study. Both studies have been described in detail and approved by a relevant Medical Ethical Committee^10,11^. RSV-specific IgG was measured in 1191 individuals, and in 497 also RSV-specific IgA was determined. Children under 1 year of age are oversampled, and the age distribution of the participant population is shown in Supplement Figure 1.

**Figure 1:**
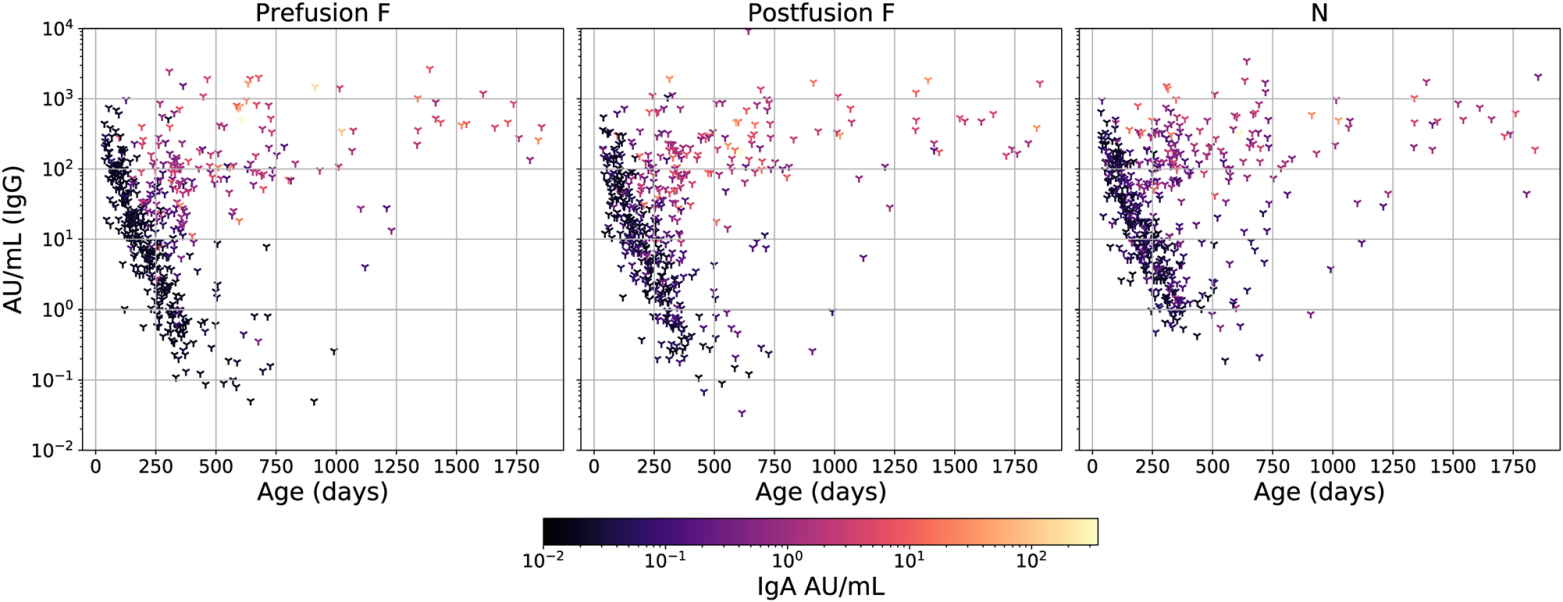
IgG and IgA concentrations as a function of age. Scatter diagram showing the IgG concentration as a function of age, and coloured by IgA concentration for three antigens, prefusion F (left-hand panel), postfusion F (middle panel), and N (right-hand panel).

A multiplex immunoassay (MIA) was performed for IgA and IgG antibody concentrations directed against five RSV antigens^13,14^. Antibody (Ab) concentrations against prefusion F protein, postfusion F protein, nucleoprotein (N), glycoprotein of RSV type A and B (Ga and Gb) were measured simultaneously. Details of the MIA are described by Schepp et al*.*^13^. Additional information from the participants is available from a questionnaire filled in by parents of the participants. The questionnaire includes information on the number and ages of household members, the number of contacts made by the participants, whether the child visits a day-care, individual characteristics (age, gestational age, weight, length), and various socio-economic factors.

For determining the timing of the RSV seasons, we use weekly data from a laboratory-based surveillance system in the Netherlands. This system surveys a selection of viral infections, including RSV^15^.

### Classification

**Figure 2:**
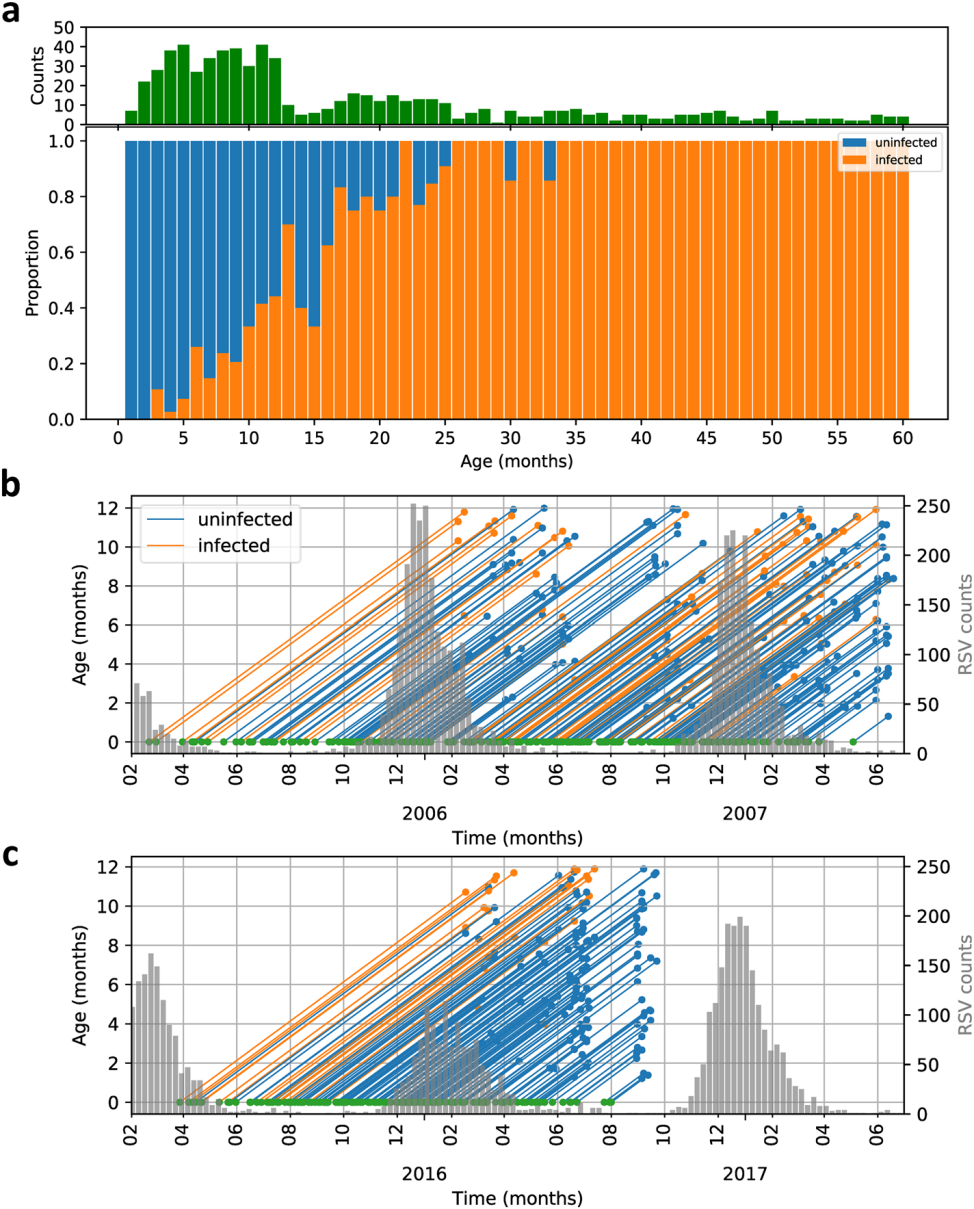
Dependence of infection status on age and date of birth within the year. (**A**) Bar chart (top) and proportional bar chart (bottom) showing the total number of samples (green), and fractions that are classified as infected (orange) and uninfected (blue). (**B**,**C**) Lexis diagrams of infection status of infants under 12 months of age in the 2006/2007 and 2016/2017 cohorts (see Supplement Figure 3 for full age range). Left-hand y-axes correspond to individual life histories. Individuals are born at the green dots, and sampled at the orange (infected) and blue (uninfected) dots, respectively. Right-hand Y-axes correspond to the histograms of the weekly number of positive RSV tests in hospitals in the Netherlands, representing the RSV epidemics.

Classification of children as previously infected (i.e. seropositive) or as yet uninfected (seronegative) is usually based on the IgG antibody concentration in a blood sample; children with Ab concentration higher than a predetermined cut-off are classified as previously infected and those with a concentration below the cut-off are classified as uninfected. Here, however, this method is complicated by the interference of maternal IgG antibodies^16^. Therefore, we have based the classification in young infants on both IgG and IgA concentrations, as IgA antibodies are not transferred across the placenta from mother to child. Specifically, in children younger than 500 days we use a previously determined IgA cut-off of 0.2 AU/mL to discriminate between infected and non-infected children^14^. For children older than 500 days an IgG cut-off of 1.0 AU/mL is used. Here, to increase specificity in both cases (i.e. under and over 500 days) we require that at least two out of three antibody concentrations (prefusion F, postfusion F and N) must exceed the cut-off for a sample to be classified as seropositive. Subgroup-specific proteins Ga and Gb are not used in the sero-classification. Using our classification method we are able to determine the infection status of 682 individuals (408 using IgA and 274 using IgG). The age distribution of the participants is shown in Fig. 2.

### Statistical model

We analyse the RSV serology using a logistic regression in a generalized additive model (GAM) using the R package mgcv^17^. The regression models express the serostatus of the participants with complete information (*n* = 616) as a function of age, birth day of year (DOY), having siblings in various age ranges, attending day-care, and sex of the individual. Continuous variables age and birth DOY are modelled using penalized cubic splines (P-splines), using second order penalties. Birth DOY is modelled with a cyclic P-spline, with the boundary knots at day 365 (December 31) attached to day 1 (January 1). Based on preliminary analyses, we supply the splines for age with 25 knots and those for birth DOY with 11 knots. In addition, having a young sibling (0–4 years) in the household and visiting day-care are modelled as categorical variables (present/absent). Parameter estimation is performed by restricted maximum likelihood. The output of the logistic regression (log-odds of infection) is transformed into a probability of prior infection using the inverse logit function. The model can be written as

$$\mathrm{log}\left(\frac{{p}_{inf}}{1-{p}_{inf}}\right)=s\left(age\right)+ cs\left(birth\, DOY\right)+ Siblings0{\text{-}}4 + day{\text{-}}care,$$

where *s*()and *cs*() represent the P-spline and cyclic P-spline, respectively.

A number of checks and supplementary analyses are performed to ensure that the results are not affected by differences between the two cohorts (2006/2007 and 2016/2017) or by specific RSV epidemics (2005/2006, 2006/2007, 2015/2016 and 2016/2017). We further exclude the potentially important covariate gestational age as only a very small number of premature infants is available (*n* = 10).

For selection of variables we perform an univariate analysis, retaining only variables that are significant at the 0.05 level (age, birth DOY, siblings0–4 and day-care) and discarding the non-significant variables (siblings5–9, siblings total, sex of the individual). Using this selection, starting from age and birth DOY, all possible model combinations are produced and checked for significance (*p* < 0.05) of the variables. In these procedures, we exclude day-care visits of household members which is marginally significant in the univariate analysis but not significant in the full model. We also evaluate all first-order interactions among variables that are significant in the univariate analyses.

## Results

### Antibody dynamics and infection status

Figure 1 shows IgG and IgA antibody concentrations as a function of age. The figure show that maternal IgG decreases at an exponential rate, such that at age 1 year the majority of samples would be classified as uninfected using the IgG cut-off. Varying the thresholds for infection appears has a minor impact on the classification (Supplement Table 1).

Figure 2 shows that the majority of participants have been infected at least once before the age of 2 years, and that all children have been infected at least once by the age of 32 months. As expected, all infected individuals have experienced one or more RSV epidemics. Supplement Figure 3 gives an overview of the results using Lexis diagrams.

### Age and birth day of year

**Figure 3:**
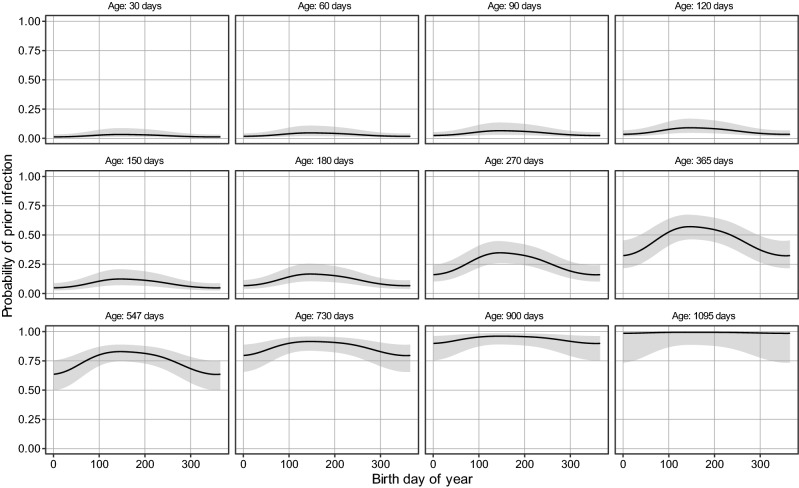
Estimates of the probability of prior infection as function of age and birth DOY. Panels show the estimated probability of prior infection as function of birth DOY within the years for various ages (baseline model). Shaded areas represent 95% confidence envelopes. See Supplement Figure 4 for additional results.

Using the logistic regression model, we find a significant effect of age and birth DOY on infection status (*p* < 2e−16 and *p* = 0.00209), while the interaction (tensor product) is not significant (*p* = 0.364). As expected, age is a major determinant for RSV infection, with probability of prior infection increasing from almost 0 at 1 month of age to virtually 1 at three years of age (Fig. 3). During the first 3 years of life, we also find a strong effect of birth DOY on the probability of prior infection. Specifically, the probability of prior infection is highest for children born in summer months (June–August) and lowest for those born in winter (December–February). For instance, at the age of 6 months, the estimated probability of prior infection for children born in July is 0.16 [95% CI 0.10, 0.24], while it is 0.07 [95% CI 0.04, 0.12] for children born in January. At the age of 1 year, the estimated probabilities of infection are 0.55 [95% CI 0.44, 0.65] for children born in July and 0.33 [95% CI 0.22, 0.47] for children born in January. Hence, at 6 months and one year of age the infection probability is 2.2- and 1.6-fold higher for children born in July as compared to those born in January. In the following, we refer to the model with age and birth DOY as the baseline model.

### Siblings and day-care attendance

**Figure 4:**
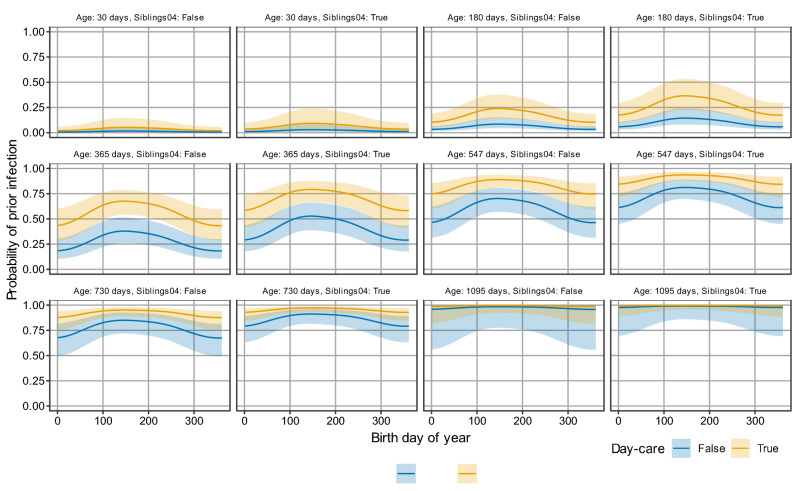
Estimates of the probability of prior infection as function of age, birth DOY, and risk factors. Shown is the estimated probability of prior infection as function of birth DOY within the year for various ages and for living with or without a young sibling (siblings0–4). Colours indicate visiting a day-care and shaded areas represent 95% confidence envelopes.

Extending the baseline model with a binary variable for having a household member in the age range 0–4 years (siblings0–4) improves the model fit significantly (*p* = 0.0364, Supplement Figure 5) and is also significant in the full model (*p* = 0.0175, Fig. 4). For example, when included in the baseline model, the estimated risk of infection at age 6 months and birth in July is 0.20 [95% CI 0.12, 0.30] for children having a young sibling (0–4 years), and 0.13 [95% CI 0.08, 0.21] for children without a young sibling. In contrast, having one or more older siblings (5–9 years) in the household does not significantly improve the model fit (*p* = 0.420; Supplement Figure 6).

Inclusion of day-care into the model strongly improves the model fit (*p* = 2.84e−06 when added to the baseline model, Supplement Figure 7; *p* = 1.58e−06 in the full model, Fig. 4). For instance, at the age of 6 months the estimated probability of infection is 0.26 [95% CI 0.16, 0.40] for children born in July that visit day-care, and 0.10 [95% CI 0.06, 0.17] for children that do not visit day-care. Noticeably, the difference between two extreme subsets (children with young siblings who visit a day-care vs children without siblings not visiting day-care) the estimated probabilities of infection at 6 months of age are 0.34 [95% CI 0.21, 0.51] and 0.08 [95% CI 0.04, 0.14], respectively, a more than fourfold difference. At 12 months of age these estimates are 0.78 [95% CI 0.65, 0.87] and 0.36 [95% CI 0.24, 0.49], respectively, still a more than twofold difference (Table 1). With increasing age, the probability of infection becomes less dependent on the day care visit and having young siblings in the household and it ceases to be a risk factor.

**Table 1:** Estimates of the probability of prior infection as function of age, birth DOY, having siblings age 0–4 and day-care visits.

Variables				Probability of prior infection		
Age (days)	Birth DOY	Siblings04	Day-care	Estimate	Lower boundary	Upper boundary
30	January 1	False	True	0.020	0.0068	0.059
30	January 1	False	False	0.0060	0.0020	0.018
30	January 1	True	True	0.037	0.013	0.10
30	January 1	True	False	0.011	0.0038	0.032
30	July 1	False	True	0.050	0.017	0.14
30	July 1	False	False	0.015	0.0050	0.046
30	July 1	True	True	0.088	0.031	0.23
30	July 1	True	False	0.028	0.0092	0.080
365	January 1	False	True	0.44	0.28	0.60
365	January 1	False	False	0.18	0.10	0.30
365	January 1	True	True	0.59	0.41	0.73
365	January 1	True	False	0.29	0.18	0.44
365	July 1	False	True	0.66	0.53	0.77
365	July 1	False	False	0.37	0.25	0.50
365	July 1	True	True	0.78	0.66	0.87
365	July 1	True	False	0.51	0.37	0.65
730	January 1	False	True	0.88	0.76	0.94
730	January 1	False	False	0.68	0.50	0.82
730	January 1	True	True	0.93	0.84	0.97
730	January 1	True	False	0.79	0.63	0.89
730	July 1	False	True	0.95	0.89	0.98
730	July 1	False	False	0.84	0.71	0.92
730	July 1	True	True	0.97	0.93	0.99
730	July 1	True	False	0.91	0.81	0.96

Shown are outcomes of the full model for a representative selection of combinations of variables. For each combination the estimate, lower and upper 95% confidence interval boundary is shown.

## Discussion

Using data from two large population-based serological studies in the Netherlands, our analyses have provided risk factors and quantitative estimates for the probability of prior RSV infection. Our results show that the probability of prior RSV infection increases strongly with age but is also highly dependent on birth DOY, with highest probabilities of infection for children born in summer (Figs. 3, 4). In addition, our results show that having a young siblings in the household (0–4 years) and attending day-care increases the probability of prior RSV infection significantly. Differences between estimated infection probabilities can be substantial. For instance, at 6 months of age children that attend day-care and have a young sibling have a more than fourfold higher estimated probability of infection than children that do not visit a day-care and do not have a young sibling (0.34 vs. 0.08).

The high incidence rate in the first 2–3 years of life is in agreement with previous studies^4,6,8,9,14^, and our infection estimates at 1 and 2 years (44.1% and 84.6%) are also comparable to estimates from Finland and Kenya^18–20^. While our results show that in the first 2 years of life children born in summer are infected at a younger age, hospitalization data from the Netherlands show that the burden in the hospital is focused in children 2–3 months old that are born between August and December^21^. Hence, this implies that while the probability of prior infection is low for children born in late summer to early winter, presumably due to the presence of maternal antibodies, the severity of disease is highest in these children.

In our analyses, age is the leading predictor for prior infection, followed by birth DOY, attending day-care, and living with children aged 0–4 years. Interestingly, having a somewhat older sibling in the household (siblings 5–9 years) is not associated with an increased probability of infection. Perhaps, the difference could be explained by the increased duration of shedding^22^ and the higher incidence of infection^7^ in younger children. With respect to day-care, it should be noted that in the Netherlands many children start going to day-care already at 3 months of age, at the end of maternity leave. An increased risk for RSV hospitalization in case of day-care attendance and preschool age sibling(s) has also been reported in previous studies^23,24^. In this study, we have shown that these risk factors also apply to infection in the Dutch general population.

We discuss a number of limitations. First, our analyses are based on 616 samples with full information on covariates. This has resulted in fairly broad confidence bands for estimates, especially in the full model with all significant factors included (Fig. 4). By extension, this also implies that we may not have uncovered risk factors with relatively small impact. And due to lack of information or small stratum size we could not include potentially relevant factors such gestational age, ethnicity, and socio-economic status into the analyses. This is unfortunate, especially with regard to prematurity, which is a known risk factor for severe infection^1^. Thus, in future studies pooling of data from the current study with seroprevalence data from other countries may increase the power to detect additional risk factors. An alternative would be a longitudinal design with multiple follow-ups and comprehensive background information on relevant factors.

Second, we have combined data from two studies, a cohort from 2006/2007 and another cohort from 2016/2017. Even though we did not find statistical evidence of differences in infection status (*p* = 0.0832) or antibody concentrations^14^ between the two studies, there are subtle differences in study setup and study characteristics. For instance the response rate was substantially higher in the 2006/2007 cohort as compared to the 2016/2017 cohort and the serum collection of young children (< 1 year) in the 2016/2017 cohort are all taken in the beginning of the sampling period, while for the 2006/2007 cohort sampling is performed scattered over the whole sampling period (see Supplement Figure 3). Nevertheless, we believe that our study has provided valuable quantitative estimates of infection rates in the first years of life that will help with the design of future vaccination strategies.

In summary, we have provided precise quantitative estimates of the probability of primary infection with RSV in the first years of life, and showed that next to age, birth DOY, day-care attendance, and having a young sibling in the household are risk factors for infection. Our results provide support for the design and prioritization of intervention and prevention measures aimed at protecting infants in the first years of life. For instance, based on our analyses one could argue that the impact of using monoclonal antibodies in selected groups would be highest when supplied to children born in summer. Of course, in practical applications, other factors such as logistical constraints and ethical considerations will have to be factored in as well.

## Supplementary information

Supplementary Informations.

## Data Availability

Data and scripts for the data processing, statistical analysis, figures, and tables are publicly available on GitHub (github.com/Stijn-A/RSV_serology).
