# Supplementary material for: Population-based serology reveals risk factors for RSV infection in children younger than 5 years
Source: Sci Rep. 2021 Apr 26;11:8953. doi: 10.1038/s41598-021-88524-w (PMC8076290; doi:10.1038/s41598-021-88524-w)
Supplement: Supplementary file 1 — Supplementary Informations. [file 41598_2021_88524_MOESM1_ESM.docx]

**Supplementary data for ‘Population-based serology reveals risk factors for RSV infection in children younger than 5 years’**

**Supplement Table 1 | Robustness of logistic regression outcome over variation in classification of infection status.**

| **Criteria for prior infection** | | | **Outcome** | | | **Significance (p-value)** | | | |
| --- | --- | --- | --- | --- | --- | --- | --- | --- | --- |
| **Age for using IgG over IgA** | **IgA cut-off**  **(AU/mL)** | **IgG cut-off**  **(AU/mL)** | **Nr of samples in log. reg.** | **Nr infected (%)** | **Nr uninfected (%)** | **age** | **birth DOY** | **siblings04** | **day-care** |
| 500 days | 0.2 | 1.0 | 616 | 310 (50.3%) | 306 (49.7%) | <2e-16 | 0.00366 | 0.0175 | 1.58e-06 |
| 400 days | 0.2 | 1.0 | 644 | 330 (51.2%) | 314 (48.8%) | <2e-16 | 0.00584 | 0.00669 | 1.23e-05 |
| 500 days | 0.2 | 10.0 | 616 | 299 (48.5%) | 317 (51.5%) | <2e-16 | 0.00228 | 0.00418 | 4.44e-08 |
| 500 days | 0.2 | 0.1 | 616 | 326 (52.9%) | 290 (47.1%) | <2e-16 | 0.000778 | 0.0222 | 2.18e-06 |
| 500 days | 0.4 | 1.0 | 616 | 288 (46.8%) | 328 (53.2%) | <2e-16 | 0.0113 | 0.00975 | 1.02e-05 |
| 500 days | 0.1 | 1.0 | 616 | 340 (55.2%) | 276 (44.8%) | <2e-16 | 0.0165 | 0.0038 | 2.35e-06 |

**
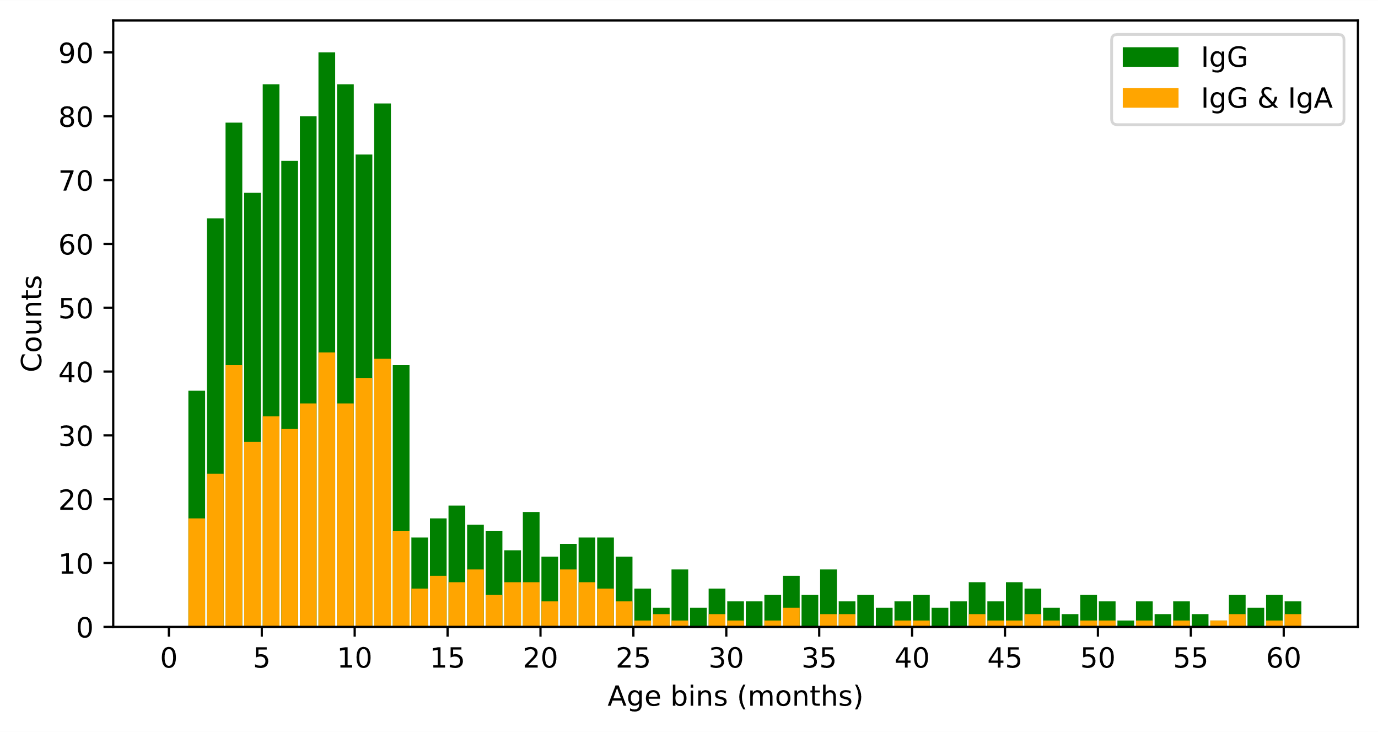
**

**Supplement Figure 1 | Stacked histogram of distribution of age (months) of children included in the study.** Samples were either tested for both IgG and IgA (yellow bars, N = 497) or for IgG only (green bars, N = 694).

**
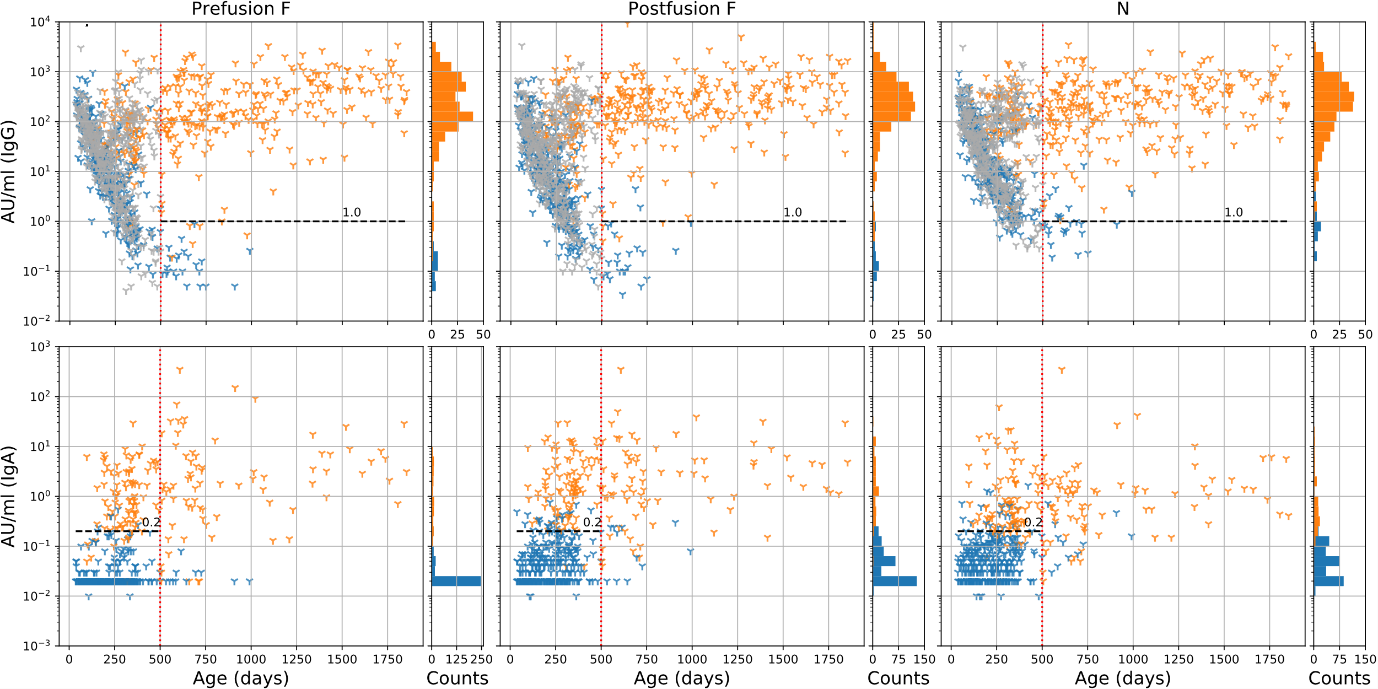
**

**Supplement Figure 2 | Classification of samples.** Shown are antibody concentrations as a function of age for the different antigens, viz. prefusion F (left-hand panels), postfusion F (middle panels) and N (right-hand panels). Top panels show IgG concentrations, and bottom panels show IgA concentration. Samples that are classified as previously infected are coloured in orange, and those that are classified as uninfected are coloured in blue. Samples that are not classified (because of missing IgA measurement) are marked in grey. The vertical histograms show the antibody distributions for samples that are classified.


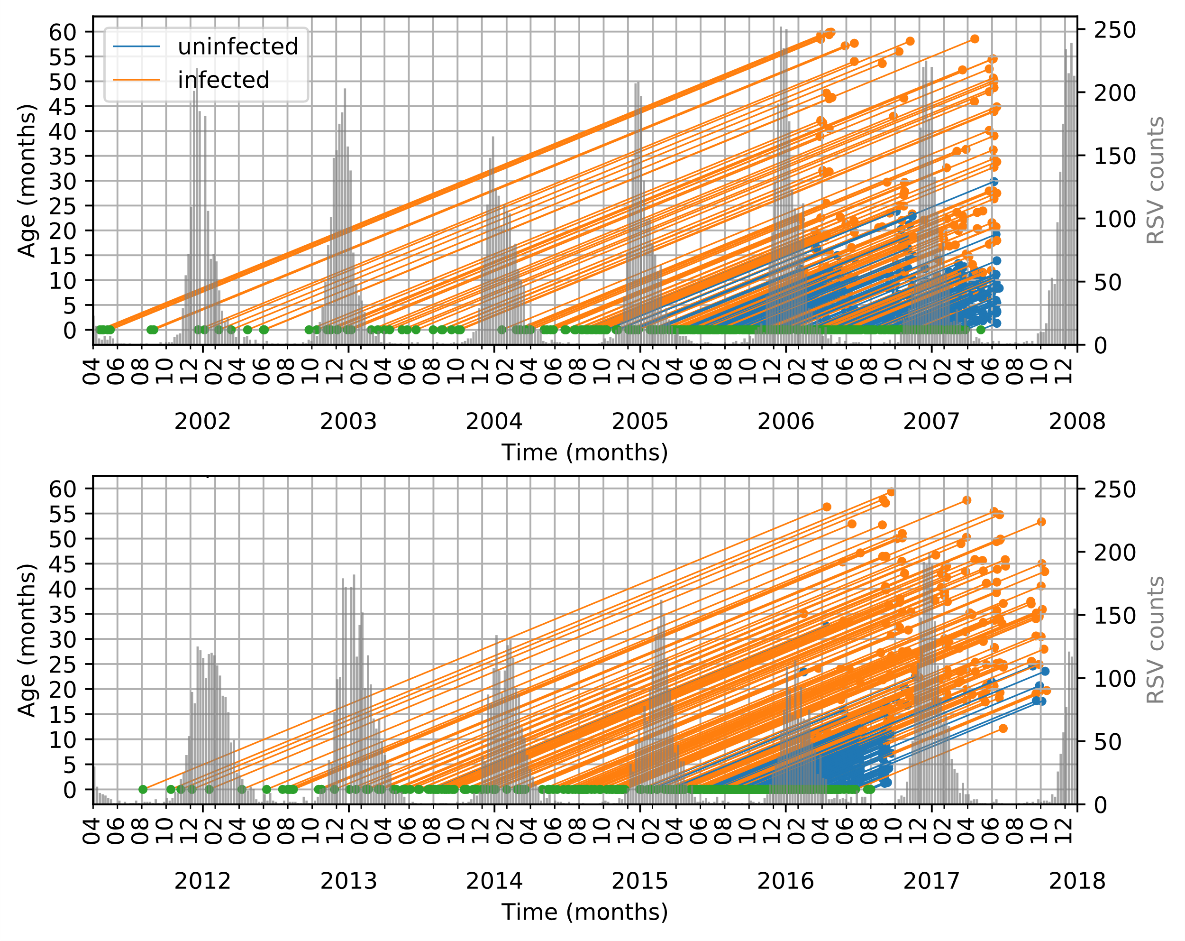


**Supplement Figure 3 | Lexis diagram of serostatus of infants and young children (age ≤ 60 months) relative to the RSV seasons.** Left-hand y-axes correspond to individual life histories (N = 682). Individuals are born at the green dots, and sampled the orange (previously infected) and blue (uninfected) dots, respectively. Right-hand Y-axes correspond to the histograms of the weekly number of positive RSV tests in the Netherlands, representing the RSV epidemics.


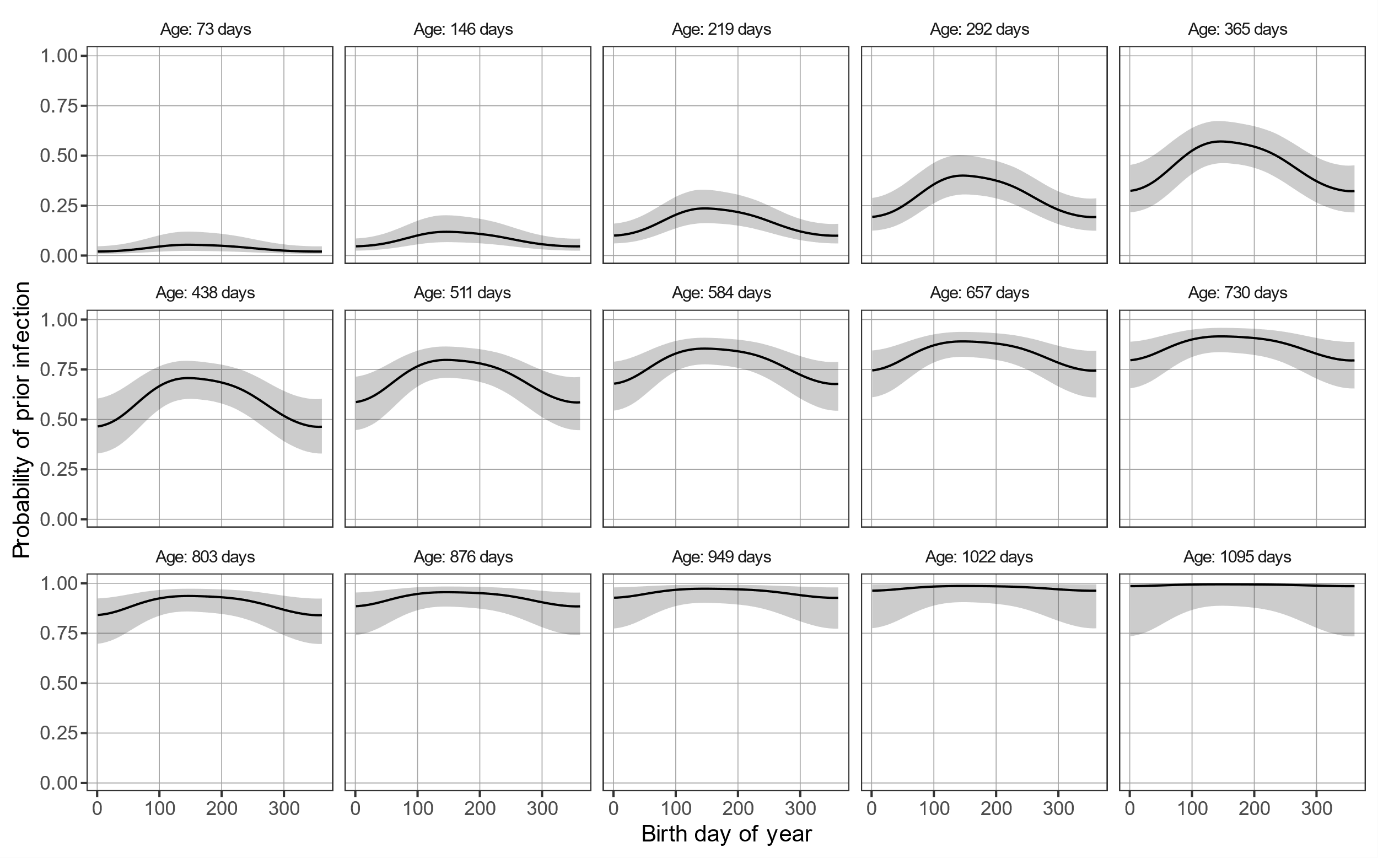


**Supplement Figure 4 | Estimation of the probability of prior infection as function of age and birth DOY.** Panels show the estimated probability of prior infection as function of birth DOY within the years for various ages. Shaded areas represent 95% confidence intervals.

**
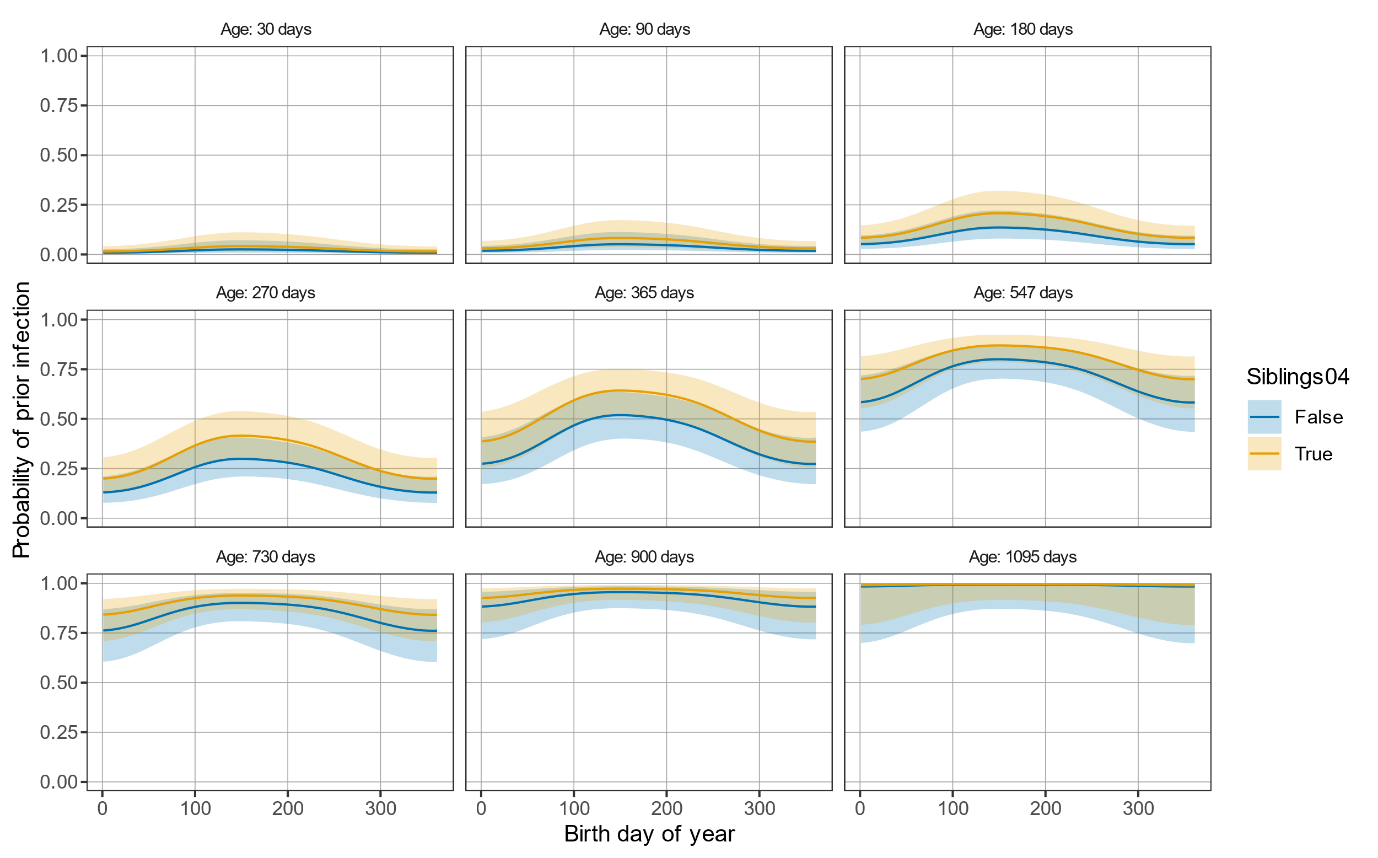
**

**Supplement Figure 5 | Estimation of the probability of prior infection as function of age, birth DOY and living with siblings (0-4 years) in the household.** Shown are the estimated probabilities of prior infection as function of birth DOY within the years for various ages and living with a housemate 0 to 4 years (Siblings04). Shaded areas represent 95% confidence intervals.

**
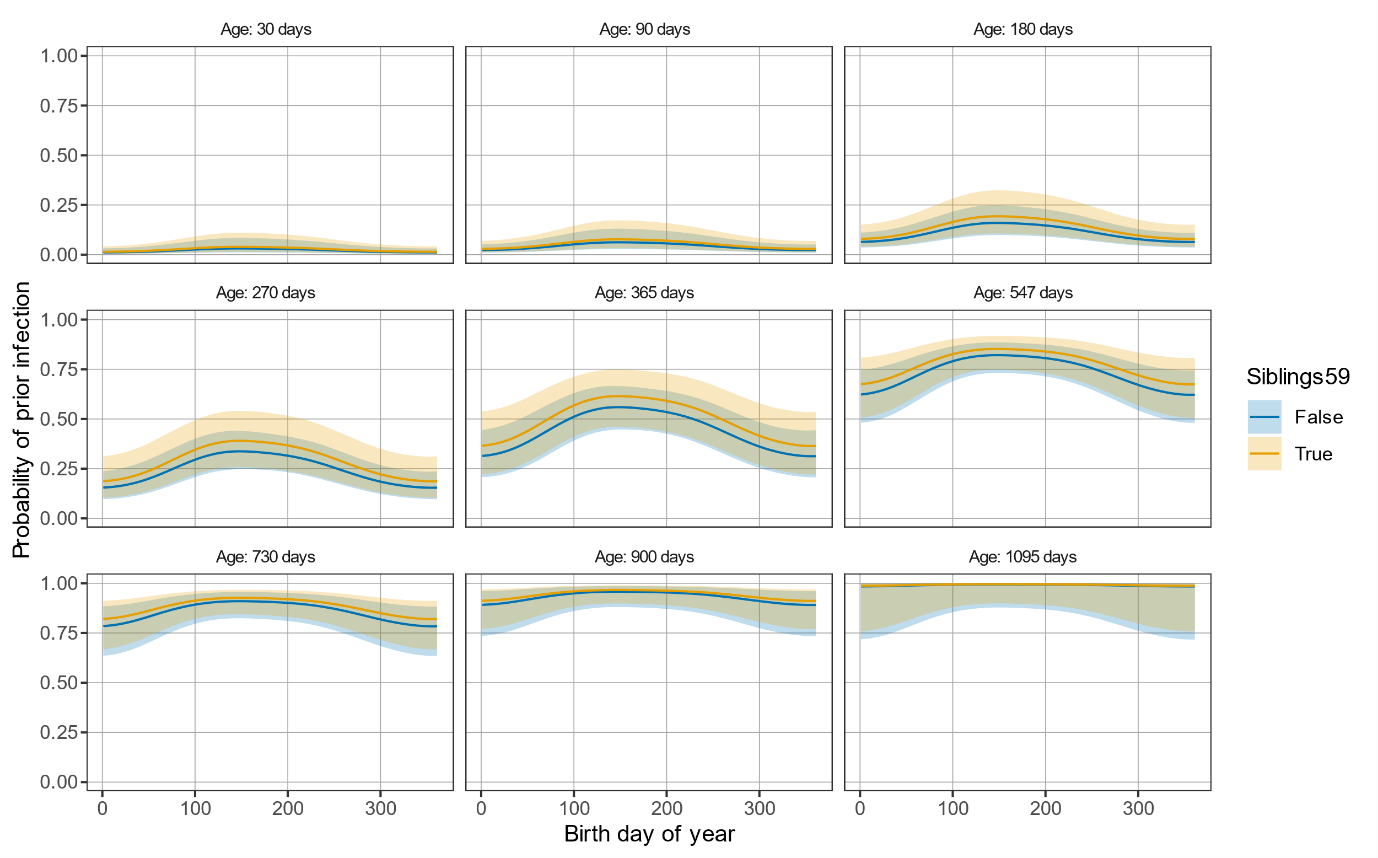
**

**Supplement Figure 6 | Estimation of the probability of prior infection as function of age, birth DOY living with siblings (5-9 years) in the household.** Shown are the estimated probabilities of prior infection as function of birth DOY within the years for various ages and living with a housemate 5 to 9 years (Siblings59). Shaded areas represent 95% confidence intervals.


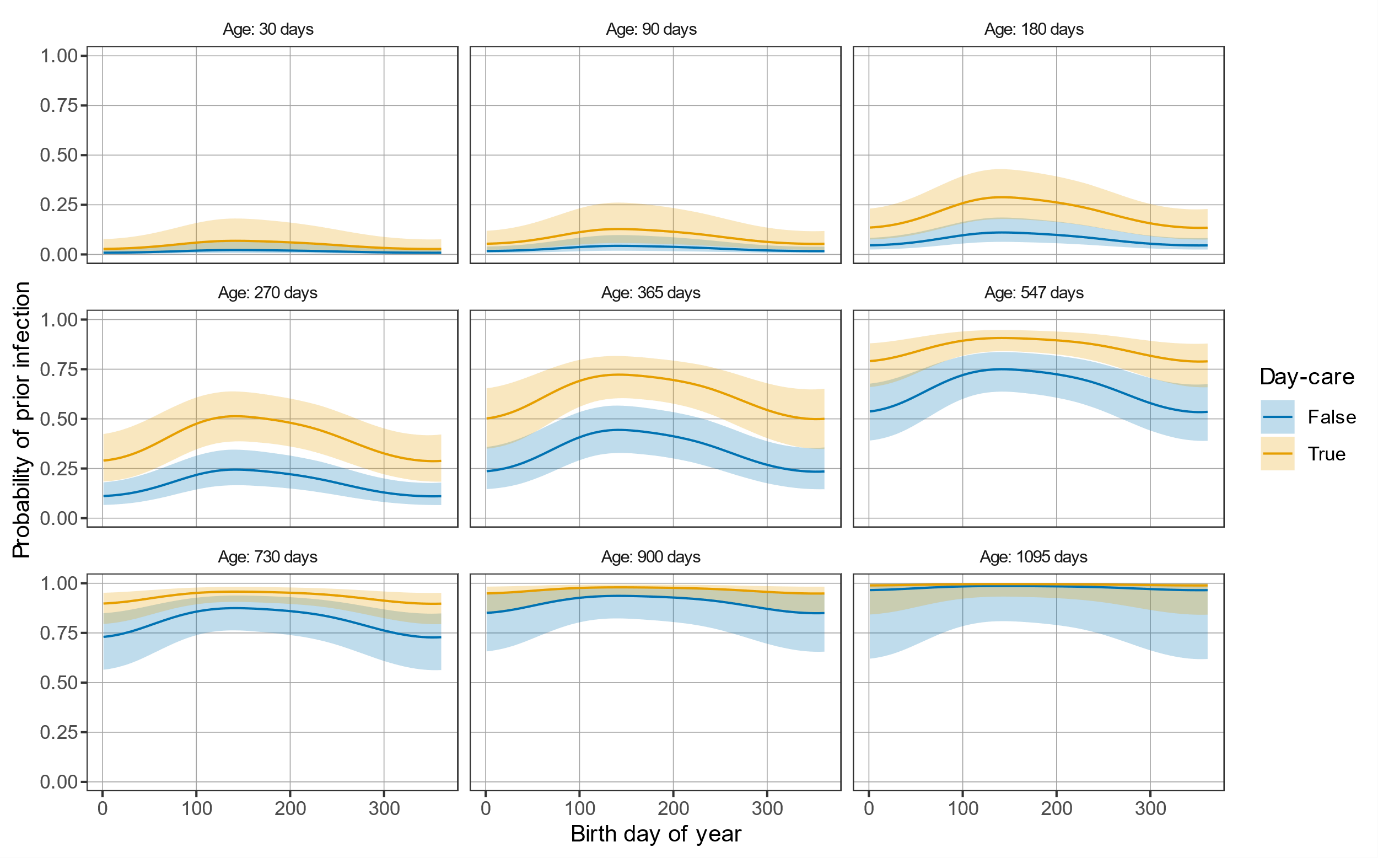


**Supplement Figure 7 | Estimation of the probability of prior infection as function of age, birth DOY and visiting a day-care.** Shown are the estimated probabilities of prior infection as function of birth DOY within the years for various ages and as function of day-care attendance. Shaded areas represent 95% confidence intervals.
